# Correlates of Sleep Disturbance Among Peoples Living in Jeju Island, Korea

**DOI:** 10.1192/bjo.2025.10163

**Published:** 2025-06-20

**Authors:** Moon-Doo Kim

**Affiliations:** Department of Psychiatry, Jeju National University Hospital, Jeju, Korea, Republic of

## Abstract

**Aims:** Individuals dissatisfied with their sleep are more likely to seek medical help, to report daytime impairment functioning, and to be diagnosed with a sleep or a mental disorder However, none of the previous studies has examined the relative importance of the various factors correlated to sleep disturbance. This study aims to investigate the prevalence of sleep disturbance and to find the associated factors contributing to sleep disturbance in the general population of Jeju Island, the largest island in the part of South Korea.

**Methods:** Seven hundred and thirteen people who consented to participate in this study and completed questionnaires were analysed. The questionnaires were used to assess the participants` sleep satisfaction and general characteristics (sex, age, marital status, occupation, monthly household income, self-perceived health, smoking, drinking status, etc.); in addition, for the clinical evaluation, depression was assessed through the Center for Epidemiologic Studies Depression Scale (CES-D) and social support through Functional Social Support Questionnaire (FSSQ). CES-D cutoff score of 21 was used to define depressive disorder. The collected data were analysed using t-test, chi-square test and logistic regression analysis according to data properties and the purpose of analysis.

**Results:** In 713 subjects, the mean age was 58.6±17.3 years, and overall, 24.9% of the subjects reported having sleep disturbance. The prevalence of sleep disturbance was higher in women than in men (60.9% vs 39.1%, crude OR=1.49, 95% CI=1.05–2.12, *p*=0.028) and increased with age (crude OR=1.03, 95% CI=1.02–1.04, *p*<0.001). The multiple logistic regression analysis demonstrated that the associated factors for the sleep disturbance were age (adjusted OR=1.04, 95% CI=1.02–1.07, *p*=0.001), smoking (adjusted OR=2.54, 95% CI=1.33–4.86, *p*=0.005) and depressive symptoms (adjusted OR=6.08, 95% CI=3.47–10.64, *p*<0.001).

**Conclusion:** Sleep disturbance was related to increasing age, smoking, and more depressive symptoms. The sleep symptoms are often unresolved by treatment, and confer a greater risk of depression. Previous epidemiological studies have pointed out that sleep problem is a risk factor for depression. There is, therefore, a need for more successful management of sleep disturbance, in order to improve quality of life and reduce an important factor in depression.

